# An Exploratory Study of Auditory Brainstem Responses and Hearing Thresholds in Essential Tremor

**DOI:** 10.3390/medicina62030495

**Published:** 2026-03-05

**Authors:** Hatice Yelda Yıldız, Mete İşeri, Pervin İşeri

**Affiliations:** 1 Department of Neurology, Faculty of Medicine, Istinye University, 34396 Istanbul, Türkiye; 2 Department of Neurology, Faculty of Medicine, Kocaeli University, 41001 Kocaeli, Türkiye; mete.iseri@gmail.com; 3 Ear, Nose and Throat Diseases Academy Group (Private Clinic), 41040 Kocaeli, Türkiye; pervin.iseri@gmail.com

**Keywords:** essential tremor, medium latency auditory evoked potentials (MLAEP), brainstem auditory evoked potentials (BAEP), hearing loss, audiometry

## Abstract

*Background and Objectives*: Essential tremor (ET) is the most prevalent movement disorder, yet its neurophysiological basis remains incompletely understood. Emerging evidence indicates that ET may involve non-motor manifestations, including auditory dysfunction. Given the anatomical convergence of tremor-related and auditory pathways at the brainstem level, electrophysiological assessment of the auditory system may provide insights into ET pathophysiology. This study aimed to evaluate auditory pathway function in patients with essential tremor using conventional audiometry, brainstem auditory evoked potentials (BAEP), and medium-latency auditory evoked potentials (MLAEP), and to examine their associations with tremor characteristics. *Materials and Methods*: Thirty patients with ET (mean age 56.6 ± 19.2 years; 15 women) and 30 healthy controls with similar age and sex distribution underwent pure-tone audiometry, BAEP, and MLAEP recordings. Tremor severity and distribution were assessed using a standardized evaluation based on the Fahn–Tolosa–Marin Tremor Rating Scale. *Results*: Conventional audiometry demonstrated normal hearing thresholds in 63.3% of ET patients and 83% of controls, while sensorineural hearing loss was observed in 36.6% and 16.6%, respectively (*p* > 0.05). High-frequency hearing loss (HFHL) was significantly more prevalent in the ET group (*p* = 0.003). BAEP analysis revealed significant prolongation of peak latencies in right-sided waves II and III and left-sided waves I and II in ET patients compared with controls (*p* < 0.05), whereas interpeak latencies (I–III, III–V, I–V) did not differ between groups. MLAEP latencies (Na, Pa, Nb) showed no significant differences between ET patients and controls (all *p* > 0.05) and were not associated with tremor severity, disease duration, or hearing asymmetry. *Conclusions*: High-frequency hearing loss is more prevalent in essential tremor, and selective BAEP latency changes observed in the context of preserved interpeak intervals suggest predominantly delayed peripheral auditory input rather than a primary brainstem conduction abnormality. Preserved MLAEP responses indicate relative sparing of thalamocortical auditory processing, supporting the concept of essential tremor as a multisystem network disorder in which altered auditory input may interact with broader network-level mechanisms.

## 1. Introduction

Essential Tremor (ET) is a neurological syndrome characterized by a slowly progressive postural and kinetic tremor that predominantly involves the upper extremities [1]. Despite being the most prevalent movement disorder worldwide, its underlying pathophysiology remains incompletely understood. Although often described as a “benign” condition, ET may worsen with advancing age and lead to substantial functional impairment, significantly affecting activities of daily living [2]. Age represents the most consistently identified risk factor for ET, with disease prevalence increasing across the lifespan and demonstrating a bimodal distribution, with peaks observed between the second and sixth decades of life [3].

The diagnosis and classification of essential tremor are primarily clinical and have evolved over time. According to the 2018 Consensus Statement of the International Parkinson and Movement Disorder Society, tremor syndromes are classified along two axes based on clinical characteristics and etiology, and essential tremor is defined within this framework as an isolated tremor syndrome [4]. Laboratory investigations and neuroimaging employed exclusively to exclude alternative or secondary causes of tremor [5]. Clinically, essential tremor is most commonly characterized by a symmetric postural and kinetic tremor involving the hands and forearms [6]. Although the tremor typically presents symmetrically at onset—most often affecting the upper limbs—it may extend to other body regions, including the head, voice, and tongue, manifesting as postural and/or action tremor [7]. The disease follows a slowly progressive course and is not associated with reduced life expectancy; however, in advanced stages, excessive tremor may markedly limit voluntary motor control, resulting in significant functional disability [8].

In recent years, ET has increasingly been conceptualized as a heterogeneous disorder, with growing recognition of a broad spectrum of non-motor manifestations [9]. Previous studies have demonstrated that hearing loss may accompany ET alongside sensorineural, psychiatric, balance, and gait-related disturbances [10,11]. Population-based screening has further indicated that approximately 30% of individuals with ET report more pronounced hearing difficulties compared with age-matched control subjects [12]. Notably, the degree of disability associated with hearing impairment in ET appears to exceed that predicted by conventional audiometric findings alone, suggesting a potential functional interaction between ET and auditory processing. Detailed audiological assessments in ET populations have consistently revealed hearing loss that is particularly prominent at high frequencies, as well as deficits in the comprehension of complex auditory stimuli, such as sentence-level speech [13]. Moreover, tremor severity has been reported to increase in parallel with the extent of hearing loss and the use of hearing amplification devices [11].

It has been proposed that a shared neural pathway underlying both hearing impairment and ET may provide insights into the pathophysiology of the disorder. However, the biological mechanisms of ET have not yet been fully elucidated and are increasingly regarded as reflecting degenerative or dysfunctional processes involving multiple interconnected neural structures and circuits rather than a single anatomical locus [14]. Accumulating evidence has implicated the cerebellum in the pathogenesis of ET, as suggested by the marked reduction in tremor following cerebellar lesions and the transient improvement of cerebellar signs after alcohol consumption [15]. Subsequent functional neuroimaging studies using positron emission tomography (PET) and functional magnetic resonance imaging (fMRI) have further supported cerebellar involvement; nevertheless, kinematic analyses of tremor have emphasized that cerebellar contributions may primarily reflect abnormalities in feedback control mechanisms rather than constituting the primary tremor generator [5,16]. In line with this concept, PET studies have demonstrated cerebellar hyperactivity in patients with ET, suggesting a modulatory role for the cerebellum within a broader tremor network [17].

Experimental models have also informed current hypotheses regarding ET pathophysiology. Harmaline-induced tremor has been widely employed as an experimental paradigm; however, it represents only a partial and imperfect analogue of human essential tremor [3,18]. In parallel, the suppression of tremor following surgical lesioning of the ventral intermediate nucleus of the thalamus has highlighted the contribution of thalamocortical pathways to tremor propagation [19,20]. It has further been suggested that tremor-related oscillatory activity may be transmitted through vestibulospinal, reticulospinal, and rubrospinal pathways, with dysfunction of a central oscillator located within the Guillain–Mollaret triangle potentially playing a contributory role [21].

Importantly, several neural structures implicated in tremor-related networks—including the inferior olive, cerebellar nuclei, pontine nuclei, and medullary brainstem pathways—are anatomically and functionally interconnected with components of the central auditory system at the level of the brainstem [14,17,21]. The olivocerebellar system, in particular, shares close neuroanatomical proximity and reciprocal connectivity with auditory brainstem nuclei, providing a plausible substrate for interaction between tremor-related oscillatory networks and auditory processing circuits. However, direct electrophysiological studies examining this potential intersection remain limited, and the functional relevance of such convergence in essential tremor has not been clearly established. Against this background, the present study was designed to explore whether measurable electrophysiological alterations within the auditory pathway are detectable in patients with ET and whether these alterations are associated with clinical tremor features. To this end, conventional audiometry, medium-latency auditory evoked potentials, and brainstem auditory evoked potentials were utilized to provide an integrated and exploratory evaluation of auditory function in ET.

The present study was designed as an exploratory investigation comparing patients with clinically defined essential tremor to age- and sex-matched neurologically healthy controls in order to determine whether measurable deviations from a normal neurophysiological baseline could be detected. Because electrophysiological studies in ET remain limited and heterogeneous, establishing differences relative to a healthy reference group was considered a necessary first step before attempting disease-specific comparisons with other neurological disorders. Inclusion of additional neurological comparison groups may further clarify the specificity of these findings; however, this was beyond the scope of the present study and should be addressed in future research.

## 2. Materials and Methods

### 2.1. Study Population

The study comprised 30 consecutive patients (15 women and 15 men) who presented to the Movement Disorders Outpatient Clinic of the Department of Neurology, Kocaeli University Faculty of Medicine, between 2006 and 2007 and were diagnosed with ET according to the Essential Tremor Diagnostic Criteria (2000) proposed by the Movement Disorder Society. Patients reporting subjective hearing impairment or presenting with additional neurological, audiological, or systemic conditions—other than ET—that could potentially affect hearing were excluded. Although the data were collected between 2006 and 2007, the electrophysiological techniques and audiometric assessment protocols used in this study remain standardized and consistent with current clinical neurophysiology practice. The more recent 2018 MDS Consensus Statement, which introduced the concept of “ET plus,” was not available during the study period. Therefore, systematic classification of patients according to contemporary ET and ET-plus criteria was not possible.

The control group was randomly selected from individuals without a genetic relationship to the patient group and was chosen to be similar in age and sex distribution. The control group was frequency-matched to the essential tremor group in terms of mean age and sex distribution in order to minimize potential age-related confounding effects on audiometric and electrophysiological parameters. All control participants had no history of audiological complaints or neurological or systemic disorders known to influence auditory function.

The inclusion of a neurologically healthy control group was intended to provide a normative reference for electrophysiological and audiometric parameters and to determine whether patients with clinically defined essential tremor exhibit measurable deviations from a normal neurophysiological baseline. The study was designed as an exploratory comparison and was not originally structured to evaluate disease specificity relative to other neurological disorders. Therefore, conclusions regarding differences between essential tremor and other movement disorders cannot be drawn from the present design.

### 2.2. Brainstem Auditory Evoked Potentials (BAEP) Recordings

BAEP recordings were obtained using an ipsilateral mastoid–Cz montage, with electrodes positioned to assess both hemispheres (active, reference, and ground). Auditory stimulation consisted of 90 dB click stimuli. Signals were band-pass filtered between 100 and 3000 Hz, and 1000 responses were averaged over a 10 ms analysis window. Absolute latencies of waves I through V and interpeak intervals (I–III, I–V, and III–V) were measured. For each ear, absolute latencies of waves I through V and interpeak intervals (I–III, I–V, and III–V) were analyzed separately. Abnormality was defined for each individual parameter as a value exceeding two standard deviations above the mean of the corresponding parameter in the age- and sex-matched control group. Absolute latency prolongation and interpeak interval prolongation were evaluated independently. Side-specific comparisons were performed for right and left ears separately. This approach allowed identification of both global latency shifts and selective abnormalities in specific components of the auditory brainstem response.

Because device-specific normative datasets were not available for the recording system used in this study, abnormality thresholds were defined relative to the internal control group distribution to ensure methodological consistency between groups. Bilateral BAEP measurements were evaluated separately for each ear, and abnormality classifications were based on side-specific comparisons with the corresponding control values.

### 2.3. Medium-Latency Auditory Evoked Potentials (MLAEP) Recordings

MLAEP recordings were performed in a dimly lit, sound-attenuated room within the Neurology Department of Kocaeli University. Participants remained awake with their eyes closed throughout the recording session. After standard skin preparation, Ag/AgCl disc electrodes were placed at the vertex and bilateral mastoids.

Auditory stimulation consisted of monaural clicks (and binaural stimulation when required) at 80 dB intensity and 0.1 ms duration, with a −40 dB contralateral masking noise and a repetition rate of 10 Hz. Signals were filtered between 100 and 3000 Hz, and 500 responses were averaged over a 100 ms epoch. The Po, Pa, and Pb peaks and the corresponding negative components (No, Na, Nb) were visually identified, and their latencies were measured in milliseconds. For each ear, Na, Pa, and Nb latencies were evaluated separately. A latency value exceeding two standard deviations above the corresponding control mean, or absence of a clearly identifiable waveform, was classified as pathological. All electrophysiological recordings were performed using a Nihon Kohden Neuropack MEB-9204K system.

As with BAEP parameters, MLAEP abnormality thresholds were determined using the internal control group distribution due to the absence of device-specific normative reference values for the recording configuration applied in this study.

### 2.4. Audiometric Examinations

Audiometric evaluations were performed in the Audiology Laboratory of the Department of Otorhinolaryngology following a brief clinical history. The assessment protocol included tympanometry, acoustic reflex testing, and conventional pure-tone audiometry across frequencies ranging from 250 to 8000 Hz. Mean hearing thresholds were calculated for the overall frequency range (250–8000 Hz) and for high frequencies (3000, 4000, and 8000 Hz), which were used to define high-frequency hearing loss (HFHL).

Hearing loss severity was classified according to the criteria proposed by Esmer et al. [22] as normal (0–20 dB), mild (20–40 dB), moderate (40–60 dB), severe (60–80 dB), profound (80–100 dB), or total (≥100 dB). Bone conduction thresholds were obtained at frequencies between 500 and 4000 Hz using a mastoid vibrator, and air–bone gaps were used to differentiate conductive, sensorineural, and mixed types of hearing loss. Audiograms were plotted with frequency (Hz) on the horizontal axis and intensity (dB) on the vertical axis.

Interaural asymmetry was evaluated using the Automated Audiogram Classification System (AMCLASS), which assesses audiogram configuration, severity, lesion site, and asymmetry. According to AMCLASS criteria, asymmetry was defined as a ≥10 dB difference at three or more frequencies, ≥15 dB at two frequencies, or ≥20 dB at a single frequency within the 250–8000 Hz range.

### 2.5. Tremor Assessment Using the Fahn–Tolosa–Marin Tremor Rating Scale (FTMTRS)

Clinical evaluation was conducted using a standardized assessment protocol based on the FTMTRS [23], which quantitatively evaluates tremor amplitude, functional disability, and motor performance.

Part A evaluates tremor severity at rest, during posture, and with action or intention across multiple body regions, including the upper extremities, head, face, voice, tongue, and trunk. Tremor amplitude is rated on a 5-point ordinal scale ranging from 0 (no tremor) to 4 (severe tremor). Part B focuses on tremor-related functional disability during specific motor tasks, such as handwriting, drawing the Archimedes spiral, pouring water, drinking from a cup, and feeding. Performance is graded using the same 0–4 scale, with higher scores indicating greater impairment. Part C assesses the overall functional impact of tremor on activities of daily living, including speaking, eating, dressing, hygiene, and occupational or social functioning. Scores reflect the degree to which tremor interferes with daily activities and independence.

A total tremor score can be calculated by summing the subscores from Parts A, B, and C, providing a comprehensive quantitative measure of tremor severity and disability. In the present study, FTMTRS scores were used to characterize tremor distribution, severity, and laterality, and to explore potential associations between clinical tremor features and electrophysiological and audiological findings.

Although patients underwent detailed neurological examination at the time of evaluation, structured prospective screening for all soft neurological signs as defined in the 2018 MDS consensus (e.g., subtle dystonia or rest tremor) was not performed according to contemporary classification criteria.

### 2.6. Statistical Analysis

Statistical analyses were performed using SPSS software version 13.0 (Statistical Package for the Social Sciences). Data distribution normality was assessed using the Kolmogorov–Smirnov test. Comparisons of normally distributed numerical variables between the patient and control groups were conducted using Student’s t-test, while the Mann–Whitney U test was applied for non-normally distributed variables. Categorical variables were compared using Pearson’s chi-square or Fisher’s exact test, as appropriate. A two-tailed *p* value < 0.05 was considered statistically significant.

Given the exploratory design of the present study and the relatively small sample size, multiple statistical comparisons were performed across electrophysiological and audiometric parameters without formal correction for multiple testing. Therefore, the results should be interpreted cautiously and considered hypothesis-generating rather than confirmatory.

Categorical abnormality analyses were based on predefined thresholds (>2 SD above control mean), allowing identification of individual-level deviations that may not be reflected in group mean comparisons.

## 3. Results

### 3.1. Demographic Characteristics of Patients with Essential Tremor

A total of 30 patients diagnosed with ET were included in the study, comprising 15 women and 15 men, with an age range of 21 to 81 years. Demographic characteristics of both the essential tremor and healthy control groups are presented in Table 1. The two groups were comparable in terms of age and sex distribution. A positive family history of tremor was reported in 18 patients (60%). Disease duration ranged from 5 to 35 years, with a mean duration of 12.1 ± 8.3 years. Thirteen patients (43.3%) were not receiving pharmacological treatment at the time of evaluation, while 11 patients (36.7%) were treated with propranolol and 3 patients (10%) with primidone. The remaining 3 patients (10%) were receiving medications other than these agents.

### 3.2. Fahn–Tolosa–Marin Tremor Rating Scale Results

**Figure 1:**
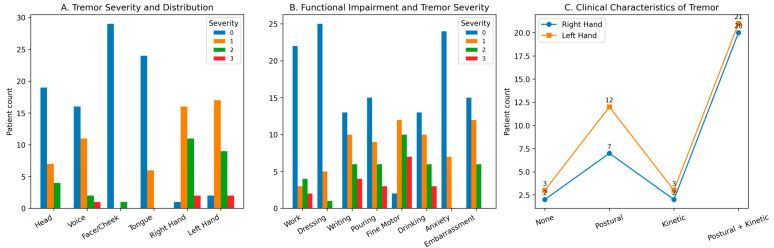
Clinical characteristics and functional impact of tremor assessed by the Fahn–Tolosa–Marin Tremor Rating Scale (FTMTRS). (**A**) shows tremor distribution across body regions according to ordinal severity scores (0–3). (**B**) presents tremor-related functional impairment in daily activities. (**C**) illustrates upper extremity tremor phenomenology. Bars represent the number of patients within each severity category. The figure is intended to display the distribution of ordinal FTMTRS scores across domains rather than summary averages.

Figure 1 presents the distribution of FTMTRS severity categories across tremor domains and functional activities, providing a descriptive overview of tremor-related clinical burden within the cohort. The FTMTRS is an ordinal scale ranging from 0 (no tremor) to 3 (severe tremor). In the present cohort, the distribution of scores corresponds predominantly to mild-to-moderate tremor severity, indicating that most patients were evaluated in relatively early or moderate stages of the disease. Tremor involvement was most frequently observed in the face/cheek region (96.7%) and tongue (80%), followed by the head (63.3%) and voice (53.3%). Upper limb tremor was also prevalent, affecting the right hand in 90% and the left hand in 83% of patients. Overall, tremor severity was predominantly mild to moderate, while severe tremor was uncommon. In the upper extremities, mild tremor was observed in 63% of right hands and 56% of left hands. Moderate tremor accounted for approximately one-third of cases, whereas severe tremor was present in fewer than 10%. With respect to tremor phenomenology, isolated postural tremor was relatively uncommon, whereas combined postural–kinetic tremor predominated (60% in the right hand and 70% in the left hand). The median tremor severity score and median Archimedes spiral score were both 1.

### 3.3. Comparison of BAEP Findings Between ET Patients and Controls

BAEP analysis demonstrated selective abnormalities in patients with ET compared with controls. Quantitative comparison of absolute peak latencies revealed significantly prolonged latencies in the ET group for right-sided wave II (2.97 ± 0.28 vs. 2.74 ± 0.27 ms, *p* = 0.002) and wave III (3.94 ± 0.27 vs. 3.73 ± 0.34 ms, *p* = 0.040), as well as left-sided wave I (2.27 ± 0.43 vs. 1.89 ± 0.48 ms, *p* = 0.001) and wave II (3.23 ± 0.39 vs. 2.96 ± 0.41 ms, *p* = 0.007) (Table 2). No statistically significant differences were observed for interpeak latencies (I–III, III–V, I–V) between the two groups.

Consistent with these latency findings, categorical analysis demonstrated a significantly higher proportion of abnormal BAEP responses in ET patients, particularly for right-sided wave II (abnormal in 60.0% of ET patients vs. 0% of controls, *p* = 0.005) and left-sided wave I (abnormal in 86.6% vs. 53.3%, *p* = 0.01). Although mean latency values for left wave V did not differ significantly between groups, a greater proportion of ET patients exhibited values exceeding the predefined abnormality threshold, resulting in a higher frequency of categorical abnormalities. (80.0% vs. 30.0%, *p* = 0.003) (Table 3). No significant group differences were identified for other BAEP components or interpeak latency abnormality rates.

### 3.4. Comparison of MLAEP Findings Between ET Patients and Controls

MLAEP analysis revealed no significant differences between patients with ET and control subjects. Mean latencies of the Na, Pa, and Nb components were comparable between groups for both right and left sides, with all comparisons yielding non-significant results (all *p* > 0.05; Table 4) Consistent with these findings, MLAEP parameters showed no association with demographic variables (age and sex), clinical characteristics (disease duration, family history, tremor type, or tremor severity), or treatment status. Additionally, MLAEP latencies did not differ between patients with shorter or longer disease duration, nor were they related to audiometric findings, BAEP abnormalities, or tremor laterality.

### 3.5. Conventional Audiometry and High-Frequency Hearing Loss in ET

Conventional pure-tone audiometry showed a higher proportion of sensorineural hearing loss in patients with ET compared with controls; however, this difference was not statistically significant. In contrast, HFHL was significantly more prevalent in the ET group (χ^2^ = 13.837, *p* = 0.003).

**Figure 2:**
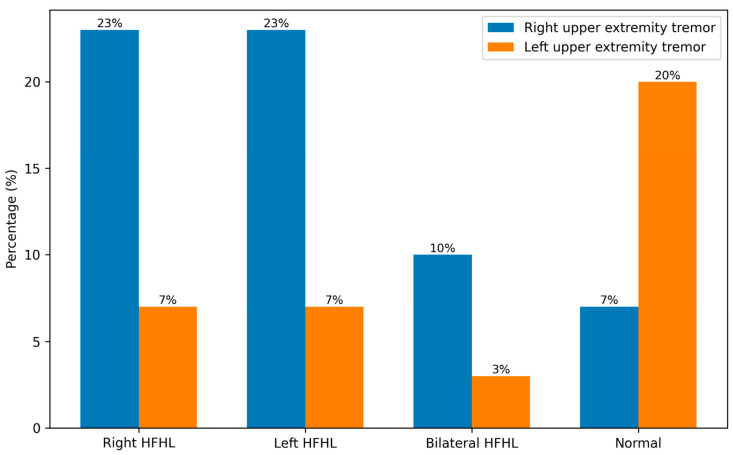
Relationship between high-frequency hearing loss and tremor laterality in essential tremor. Association between high-frequency hearing loss (HFHL) patterns and upper extremity tremor laterality in essential tremor. Values are presented as percentages.

As illustrated in Figure 2, asymmetric HFHL was markedly more frequent among ET patients, particularly left-sided asymmetry, whereas right-sided asymmetry was absent in controls. Notably, 90% of individuals with left-sided asymmetric HFHL and all individuals with right-sided asymmetric HFHL belonged to the ET group. Symmetric HFHL was observed in both groups but did not distinguish ET patients from controls.

**Figure 3:**
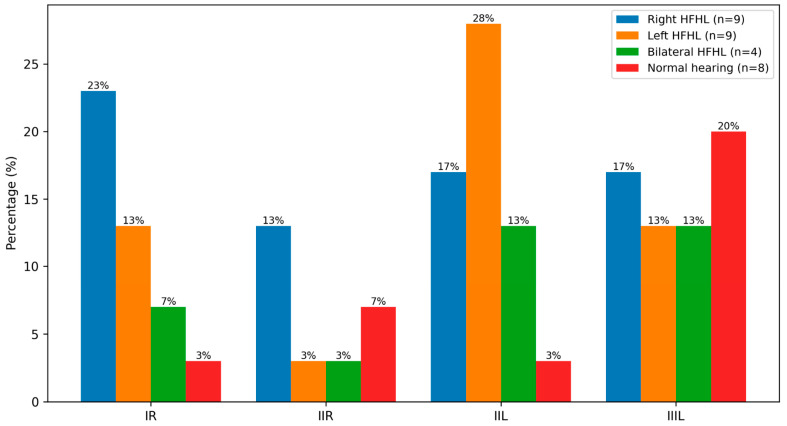
Relationship between high-frequency hearing loss and prolonged BAEP responses in patients with essential tremor. Distribution of prolonged brainstem auditory evoked potential (BAEP) responses according to high-frequency hearing loss (HFHL) categories in essential tremor. Values are expressed as percentages.

Analysis of tremor laterality demonstrated that asymmetric HFHL tended to co-occur with unilateral tremor, while symmetric HFHL was more commonly associated with bilateral tremor patterns (Figure 3). Despite this tendency, no statistically significant relationship was identified between HFHL patterns and BAEP latency prolongation. Furthermore, HFHL was not associated with MLAEP parameters, tremor severity, age, sex, disease duration, medication use, or tremor type.

## 4. Discussion

The present study aimed to investigate auditory pathway involvement in ET by integrating conventional audiometry with BAEP and MLAEP. The principal findings can be summarized as follows: (i) HFHL was significantly more prevalent in ET patients than in controls (χ^2^ = 13.837, *p* = 0.003), despite the absence of subjective hearing complaints; (ii) BAEP analysis revealed selective prolongation of early absolute wave latencies, particularly waves I–III, without corresponding interpeak latency abnormalities, specifically right-sided waves II–III and left-sided waves I–II; and (iii) MLAEP responses did not differ between ET patients and controls. Importantly, neither BAEP nor MLAEP abnormalities showed a clear relationship with tremor severity, disease duration, or medication use, suggesting subclinical auditory pathway alterations. Given the preservation of interpeak latencies, these findings may primarily reflect delayed peripheral auditory input rather than a definitive brainstem conduction abnormality.

Recent studies have increasingly emphasized the role of non-motor manifestations in essential tremor, including sensory, cognitive, and auditory features, supporting the concept of ET as a multisystem network disorder rather than a purely motor condition. Electrophysiological investigations of auditory processing in ET remain limited, and available studies have reported heterogeneous findings regarding brainstem and cortical auditory pathway involvement. Our results add to this emerging body of literature by suggesting subtle alterations in auditory processing that may originate at the peripheral or input level, while central brainstem conduction appears largely preserved and cortical-level responses remain relatively intact.

Hearing impairment has increasingly been recognized as a non-motor feature of ET. Ondo et al. reported significantly greater hearing disability and a higher prevalence of high-frequency sensorineural hearing loss in ET compared with Parkinson’s disease and healthy controls, with hearing loss correlating with tremor severity (*p* = 0.02) [12]. In contrast, although HFHL was significantly more frequent in our ET cohort (36.6% vs. 16.6% in controls), we did not observe a relationship between hearing loss and tremor severity, laterality, or disease duration. This discrepancy may reflect methodological differences, as our study focused on patients without subjective hearing complaints and relied on objective audiometric thresholds rather than hearing handicap indices.

Notably, asymmetric HFHL was common in our ET group, with left-sided asymmetry observed in 33.3% and right-sided asymmetry in 10% of patients, whereas right-sided asymmetry was absent in controls. Asymmetric HFHL tended to accompany unilateral tremor, although this association did not reach statistical significance. These findings align with community-based observations that tremor asymmetry is a fundamental property of ET, often mild to moderate in magnitude [7], and extend this concept to the auditory domain.

The observed prolongation of early BAEP wave latencies (waves I–III), particularly the delay in wave I, in the absence of significant interpeak latency differences, suggests that delayed peripheral auditory input may be the primary contributor to these findings. Preserved interpeak intervals (I–III, III–V, I–V) generally indicate intact conduction through the brainstem auditory pathways. Accordingly, the electrophysiological pattern observed in this study appears more consistent with peripheral involvement at the cochlear or auditory nerve level rather than a primary central brainstem conduction defect.

Nevertheless, early auditory structures such as the auditory nerve and cochlear nucleus are anatomically and functionally interconnected with olivopontocerebellar circuits implicated in tremor generation [14,15]. Neuroimaging studies have demonstrated medullary and thalamic metabolic alterations in essential tremor, suggesting broader network-level involvement that may include brainstem-related pathways [15]. Therefore, while the present BAEP findings do not provide direct evidence for impaired brainstem conduction, subtle interactions between peripheral auditory input pathways and tremor-related network circuits cannot be entirely excluded and should therefore be interpreted cautiously as a hypothesis-generating observation.

Interestingly, previous reports of focal brainstem lesions provide useful context. Vitte et al. described bilateral inferior collicular lesions causing word deafness with preserved BAEP responses, emphasizing that BAEP abnormalities depend on lesion location and pathway integrity [21]. Similarly, unilateral lesions of the lateral lemniscus have been associated with delayed or reduced BAEP wave V, while MLAEPs remain normal [20]. The preservation of MLAEPs in our ET cohort, despite BAEP abnormalities, supports the hypothesis that subcortical brainstem pathways are preferentially involved, whereas thalamocortical auditory processing remains relatively intact.

MLAEP components are believed to originate from bilateral thalamocortical projections and primary auditory cortices. The absence of MLAEP abnormalities in our ET patients suggests that thalamocortical auditory processing is preserved, despite evidence implicating the thalamus in tremor propagation. This finding is consistent with clinical observations that ventral intermediate nucleus (Vim) stimulation effectively suppresses tremor without altering sensory processing [19]. Together, these findings are compatible with current network models of ET involving cerebellar and thalamocortical circuits, while the present electrophysiological pattern primarily reflects peripheral auditory input alterations.

Current models conceptualize ET as a network disorder involving cerebellar, brainstem, and thalamocortical circuits rather than a single oscillator [16,17]. Experimental and neuropathological evidence highlights the role of the inferior olive and its inhibitory feedback loops with the cerebellum in tremor generation [18]. Although the present BAEP pattern does not indicate impaired brainstem conduction, early auditory structures share anatomical and functional connections with cerebellar and olivary circuits implicated in tremor generation. Therefore, altered peripheral auditory input may interact with broader tremor-related network mechanisms rather than reflecting a primary brainstem conduction defect. However, this interpretation remains speculative and should be considered hypothesis-generating rather than confirmatory.

Several limitations should be acknowledged. A major limitation of this study relates to the historical nature of the dataset. Participants were recruited between 2006 and 2007 and diagnosed according to the Movement Disorder Society 2000 criteria for essential tremor. The 2018 MDS Consensus Statement introduced revised diagnostic boundaries and the concept of “ET plus,” which includes additional neurological soft signs. Because these criteria were not available at the time of data collection, retrospective reclassification of the cohort according to contemporary standards was not feasible. Consequently, it is possible that a subset of patients included in the ET group might be classified differently under current diagnostic frameworks. This should be considered when interpreting the findings. The sample size was modest, potentially limiting statistical power for comperative analyses. An additional limitation relates to the use of the FTMTRS for tremor assessment. Although FTMTRS was widely used and accepted at the time of data collection, more recent instruments such as the Tremor Research Group Essential Tremor Rating Assessment Scale (TETRAS) provide improved sensitivity and psychometric robustness. Because the present dataset was collected between 2006 and 2007, TETRAS was not available during the study period, and retrospective reclassification using contemporary tremor scales is not feasible. Future studies employing modern tremor assessment instruments may allow more precise characterization of associations between tremor severity and electrophysiological findings. Most patients were evaluated at relatively early disease stages and did not report subjective hearing loss, which may have attenuated observed associations. Audiological assessment was restricted to conventional pure-tone audiometry; advanced measures such as otoacoustic emissions or speech-in-noise testing were not performed. In addition, the relatively large number of statistical comparisons performed without formal adjustment for multiple testing may increase the risk of type I error. Accordingly, statistically significant findings should be interpreted with caution and regarded as exploratory observations that require confirmation in larger, hypothesis-driven studies. Furthermore, although the control group was age-matched to the patient group, formal age-adjusted analyses were not performed. Given the well-established age dependence of high-frequency hearing loss and BAEP latencies, residual confounding by age cannot be entirely excluded. Additionally, abnormal electrophysiological values were defined relative to the internal control group distribution. Given the modest size of the control group, thresholds based on ±2 standard deviations may be statistically less stable and should be interpreted with caution. Future studies using larger normative datasets or external reference values would help to validate the observed abnormality rates. The pathophysiological interpretation of BAEP abnormalities in relation to olivocerebellar or tremor-related networks should be interpreted cautiously, as the present study was not designed to directly test mechanistic hypotheses. Further multimodal and longitudinal studies integrating electrophysiology with neuroimaging are needed to clarify these relationships. Additionally, the dataset was collected between 2006 and 2007. Although the electrophysiological methods employed remain valid and standardized, evolving conceptual frameworks of essential tremor and advances in multimodal neuroimaging warrant cautious interpretation of the findings in a contemporary context. An additional limitation is the absence of other neurological comparison groups. Although inclusion of patients with other movement disorders could enhance disease-specific interpretation, the present study was designed as an exploratory comparison between essential tremor and neurologically healthy individuals. Future studies incorporating multiple neurological cohorts are needed to determine the specificity of the observed auditory alterations. A further limitation relates to the potential influence of tremor medications on electrophysiological parameters. Although a proportion of patients were receiving propranolol or primidone at the time of evaluation, the cross-sectional design does not allow definitive exclusion of subtle pharmacological effects. Therefore, medication-related confounding cannot be entirely ruled out. Finally, the cross-sectional design precludes conclusions regarding causality or progression.

Future studies should incorporate larger, longitudinal cohorts and employ more sensitive auditory assessments to clarify the temporal relationship between tremor progression and auditory dysfunction. Combining electrophysiological methods with functional neuroimaging may further elucidate shared network abnormalities underlying motor and non-motor features of ET. Such integrative approaches may ultimately contribute to refined phenotyping and targeted therapeutic strategies.

Accordingly, the present findings should be interpreted within the historical diagnostic context of the cohort and regarded as exploratory observations in clinically defined essential tremor populations.

## 5. Conclusions

In conclusion, our findings demonstrate that high-frequency hearing loss is significantly more prevalent in essential tremor. Selective BAEP latency changes observed in the context of preserved interpeak intervals suggest predominantly delayed peripheral auditory input rather than a primary brainstem conduction abnormality. The preservation of MLAEP responses indicates largely intact thalamocortical auditory processing. By integrating conventional audiometry with both brainstem and medium-latency auditory evoked potentials in a clinically characterized ET cohort, the present study provides additional descriptive evidence for subtle, subclinical auditory pathway involvement in essential tremor. Although these electrophysiological changes did not show a clear relationship with tremor severity, they may reflect subclinical alterations in auditory processing that interact with broader multisystem network mechanisms in essential tremor. These findings should be interpreted cautiously and considered hypothesis-generating. Future studies using contemporary diagnostic criteria, larger and longitudinal cohorts, and multimodal neurophysiological or neuroimaging approaches are needed to confirm and further characterize these observations.

## Figures and Tables

**Table 1:** Demographic and Clinical Characteristics of the Study Population.

	Essential Tremor n (%)	Controls n (%)
N	30 (100%)	30 (100%)
Age (mean ± SD)	56.6 ± 19.2	56.6 ± 19.2
Sex		
Female	15 (50.0)	15 (50.0)
Male	15 (50.0)	15 (50.0)
Dominant side		
Right	19 (63.3)	-
Left	11 (36.7)	-
Family history		
Present	18 (60.0)	-
Absent	12 (40.0)	-
Disease duration (years)		
5–10	19 (63.3)	-
11–15	2 (6.7)	-
16–20	4 (13.3)	-
≥21	5 (16.7)	-
Medication use		
None	13 (43.3)	-
Propranolol	11 (36.7)	-
Primidone	3 (10.0)	-
Others	3 (10.0)	-

n: number of cases.

**Table 2:** BAEP Values of Essential Tremor Patients and the Control Group.

BAEP	Control Group (Mean ± SD)	Essential Tremor (Mean ± SD)	Test Statistics	*p*
**Right**				
I	1.65 ± 0.26	1.80 ± 0.33	1.79 **	0.074
II	2.74 ± 0.27	2.97 ± 0.28	3.196 **	**0.002**
III	3.73 ± 0.34	3.94 ± 0.27	2.056 *	**0.040**
IV	4.98 ± 0.50	5.16 ± 0.33	1.57 **	0.123
V	5.75 ± 0.47	5.81 ± 0.37	0.902 *	0.367
**Left**				
I	1.89 ± 0.48	2.27 ± 0.43	3.232 *	**0.001**
II	2.96 ± 0.41	3.23 ± 0.39	2.677 *	**0.007**
III	4.03 ± 0.59	4.36 ± 0.42	1.908 *	0.056
IV	5.09 ± 1.04	5.44 ± 0.54	1.708 *	0.088
V	6.26 ± 1.15	6.61 ± 0.66	1.42 *	0.161
**Interpeak latencies**				
I–III	2.08 ± 0.33	2.12 ± 0.29	0.584 **	0.561
III–V	2.10 ± 0.37	1.86 ± 0.20	1.909 *	0.056
I–V	4.10 ± 0.52	3.98 ± 0.40	0.362 *	0.717

* Mann–Whitney U test. ** Student’s *t* test.

**Table 3:** Comparison of BAEP Pathologies in Essential Tremor Cases with the Control Group.

BAEP	Essential Tremor n (%)	Control Group n (%)	*p*
Right			
I			
Normal	14 (46.6)	19 (63.3)	>0.05
Abnormal	16 (53.3)	11 (36.6)	
II			
Normal	12 (40.0)	30 (100.0)	0.005
Abnormal	18 (60.0)	0 (0.0)	
III			
Normal	25 (83.3)	29 (96.6)	>0.05
Abnormal	5 (16.6)	1 (3.3)	
IV			
Normal	28 (93.3)	28 (93.3)	>0.05
Abnormal	2 (6.6)	2 (6.6)	
V			
Normal	28 (93.3)	27 (76.6)	>0.05
Abnormal	2 (6.6)	3 (23.3)	
Left			
I			
Normal	4 (13.3)	14 (46.6)	0.01
Abnormal	26 (86.6)	16 (53.3)	
II			
Normal	7 (23.3)	14 (46.6)	>0.05
Abnormal	23 (76.6)	16 (53.3)	
III			
Normal	9 (30.0)	14 (46.6)	>0.05
Abnormal	21 (70.0)	16 (53.3)	
IV			
Normal	18 (70.0)	19 (63.3)	>0.05
Abnormal	12 (30.0)	11 (36.6)	
V			
Normal	6 (20.0)	18 (70.0)	0.003
Abnormal	24 (80.0)	12 (30.0)	
Interpeak Latencies			
I–III			
Normal	27 (90.0)	26 (98.6)	>0.05
Abnormal	3 (10.0)	4 (1.3)	
III–V			
Normal	30 (100.0)	25 (83.3)	0.052
Abnormal	0 (0.0)	5 (16.6)	
I–V			
Normal	29 (96.6)	25 (83.3)	>0.05
Abnormal	1 (3.4)	5 (16.6)	

n: number of cases.

**Table 4:** Comparison of Medium-Latency Auditory Evoked Potential (MLAEP) Latencies Between Essential Tremor Patients and Controls.

Side	MLAEP Component	Control Group (Mean ± SD)	ET group (Mean ± SD)	Test Statistics	*p*
**Right**	Na	13.29 ± 2.07	12.44 ± 1.78	1.488	0.137
	Pa	17.76 ± 2.60	16.89 ± 1.84	1.490	0.142
	Nb	23.65 ± 3.40	23.47 ± 3.29	0.206	0.838
**Left**	Na	12.88 ± 2.22	12.60 ± 1.77	0.522	0.604
	Pa	17.15 ± 2.75	17.12 ± 1.58	0.466	0.641
	Nb	22.84 ± 2.20	22.63 ± 3.33	0.526	0.599

## Data Availability

The data supporting the findings of this study are available from the corresponding author upon reasonable request.

## Abbreviations

The following abbreviations are used in this manuscript:

AMCLASS	Automated Audiogram Classification System
BAEP	Brainstem Auditory Evoked Potentials
dB	Decibel
ET	Essential Tremor
FTMTRS	Fahn–Tolosa–Marin Tremor Rating Scale
fMRI	Functional Magnetic Resonance Imaging
HFHL	High-Frequency Hearing Loss
Hz	Hertz
MLAEP	Medium-Latency Auditory Evoked Potentials
ms	Millisecond
PET	Positron Emission Tomography
SD	Standard Deviation
